# Facilitators to the implementation of exercise into cancer care: a systematic review

**DOI:** 10.1007/s11764-025-01948-5

**Published:** 2026-01-04

**Authors:** Georgia L. White, Mary A. Kennedy, Brent J. Cunningham, Riley Dunn, Jason Martin, Meegan Walker, Craig T. Wallington-Gates, Hattie Wright, Bryan Chan, Grace L. Rose

**Affiliations:** 1 University of the Sunshine Coast, Sippy Downs, QLD 4556 Australia; 2 Nutrition and Health Innovation Research Institute, Edith Cowan University, Joondalup, WA Australia; 3 Queensland University of Technology, Brisbane, QLD Australia; 4 Cancer Council Queensland, Brisbane, QLD Australia; 5 Sunshine Coast University Hospital, Birtinya, QLD Australia; 6 Griffith University, Southport, QLD Australia; 7 Sunshine Coast Health Institute, Birtinya, QLD Australia; 8 The University of Queensland, Brisbane, QLD Australia

**Keywords:** Cancer, Physical activity, Enablers, Clinical practice

## Abstract

**Purpose:**

Although governing bodies urge the importance of integrating exercise into routine cancer care, facilitators to implementation have not been systematically identified and collated. This review aimed to summarise facilitators to implement exercise services within real-world settings.

**Methods:**

A systematic review of qualitative, quantitative, and mixed-method studies was conducted (three databases, 13th September 2024). Reports were included if they documented the implementation of co-located exercise services (delivered onsite within cancer centres) or referral pathways to external exercise services from cancer centres. The updated Consolidated Framework for Implementation Research (CFIR 2.0) was used to code and synthesise facilitators.

**Results:**

Of 8544 search results, 28 reports (full-text articles) describing 29 implementation efforts (structured initiatives to integrate an exercise service within a cancer hospital or centre) were included. One hundred and sixty-four facilitators were identified across all CFIR 2.0 domains. Facilitators existed within all CFIR 2.0 domains; inner setting (30%), implementation process (27%), innovation (21%), individuals (15%), and outer setting (7%). The most frequent CFIR 2.0 constructs identified were (i) access to knowledge and information, (ii) engaging recipients and deliverers, (iii) individual motivation, (iv) innovation adaptability, (v) reflecting and evaluating on the innovation and implementation.

**Conclusions:**

The implementation of exercise services within cancer settings is influenced by an interconnected network of facilitators, with an organisation’s resources and willingness playing a central role. This review provides a generalised blueprint of facilitators and considerations to guide stakeholders in their own exercise oncology contexts.

**Implications for Cancer Survivors:**

This map of facilitators provides potential opportunities for clinicians, policy makers, and researchers, to advance efforts to embed exercise in routine cancer care, so that all people living with or beyond cancer have the opportunity to benefit from it.

**Supplementary Information:**

The online version contains supplementary material available at https://doi.org/10.1007/s11764-025-01948-5.

## Introduction

Globally, there were almost 20 million new cancer cases in 2022 [1]. This number is forecast to reach 35 million by 2050, which from now, reflects a 77% increase of people diagnosed with cancer [1]. Physical and mental repercussions of cancer and its treatments are common, and result in significant health impacts throughout survivorship, individually and across wider society [2–5]. Exercise has a clear role in cancer care, with evidence for cancer prevention [6], enhanced survival [6, 7], and management of both physical and mental effects of treatment (i.e., fatigue, physical function, anxiety, depression) [8–10]. Based on this evidence, position statements and guidelines urge the importance of exercise for all people living with or beyond cancer [8, 11–14].

Governing authorities, including the Clinical Oncology Society of Australia (COSA) and the American College of Sports Medicine (ACSM), also call for all cancer care clinicians and other key stakeholders (e.g., researchers, policy makers) to take part in implementing exercise within routine clinical practice [12, 14]. Despite these position statements and urgent calls for action, the routine integration of exercise into cancer practice is lacking. Only 58% of health professionals, including oncologists and nurses, self-report discussing exercise with their patients [15], with the average referral rate to an exercise professional or service below 20% [15]. Furthering this, only 16% of people living with or beyond cancer meet physical activity guidelines during treatment, which increases to 37% post-treatment [15].

Unlike traditional research, such as randomised control trials, where contextual factors are highly controlled, implementation science understands any real-world context is complex, where many determinants (barriers and facilitators) can help or hinder implementation [16, 17]. Multiple barriers to the successful implementation of exercise in oncology practice have been comprehensively documented and summarised [18]. These barriers include healthcare professionals’ lack of knowledge and attitudes towards exercise, limited time to discuss exercise in appointments, poorly established referral pathways and networks, low perceived advantages in practice, reduced accessibility (e.g., cost, availability, transport), and minimal resources (e.g., funding, equipment, qualified staff) [18]. Ultimately, these known barriers to exercise implementation limit access to exercise for people living with or beyond cancer. Although some studies have identified what assists the integration of exercise within cancer centres [19–21], there is currently no global “knowledge bank” of such facilitators. While a review by Czosnek et al. [22] synthesised implementation outcomes of exercise oncology programs (acceptability, adoption, appropriateness, cost, feasibility, fidelity, penetration and sustainability), there remains a gap in understanding determinants that influence successful implementation. Comprehensively understanding facilitators to exercise in cancer care is an important starting point to begin organisation-specific exercise implementation processes. Therefore, this systematic review aims synthesise global facilitators that assist the integration of exercise oncology services within cancer care centres, from quantitative, qualitative, and mixed methods studies.

## Methods

### Design

A systematic review (Open Science Framework ID: osf.io/exw46) was conducted in accordance with the Preferred Reporting Items for Systematic reviews and Meta-Analyses (PRISMA) 2020 [23] (Supplementary Table 1).

### Definitions

Several definitions were operationalised for this review, as shown in Table 1*.*

**Table 1:** Definitions operationalised within the review

Term	Definition within this review
Facilitators	“Things” that predict or explain actual implementation outcomes [24]. Due to the nascency of the exercise oncology implementation field and lack of consistent terminology applied within the literature, implementation strategies were also considered facilitators As recommended by Damschroder et al. when using the updated Consolidated Framework for Implementation Research (CFIR 2.0), data related to facilitators (quantitative or qualitative) is best collected from people that have influence and authority over implementation outcomes and are commonly involved in the implementation and/or delivery of the exercise service [24]. The CFIR 2.0 should not be utilised to gather data directly from recipients (people living with or beyond cancer), unless they helped to implement and/or deliver the innovation [24]. Instead, recipient experiences with the exercise service explain the outcomes, which is outside of this review’s scope. Therefore, data reported by recipients of exercise services (people living with or beyond cancer) who were not implementation team members, were not considered as facilitators within this review [24].
Implementation outcomes	The effects of deliberate and purposive actions to implement the exercise service (e.g., adoption, acceptability, reach, effectiveness, cost, maintenance) [25]
Implementation effort	A structured process of integrating an exercise service within a cancer hospital/centre [26]
Report	A report is defined as the document outlining the results, which may describe singular or multiple implementation efforts (i.e., the process of implementing the exercise service)
Exercise service	A program or clinic designed to deliver exercise to people living with or beyond cancer. The deliverer of the exercise service may be, but not limited to, exercise physiologist, physiotherapy, personal trainer, nurse
Co-located exercise service	An exercise service delivered within the same setting as the cancer centre/hospital
Referral pathway	A process which people living with or beyond cancer are referred from the cancer centre/hospital to an exercise service delivered outside the cancer centre/hospital

#### Search strategy

Searches were conducted by GLW across three databases (PubMed, Scopus, and CINAHL) on 13th September 2024. Search terms included free text terms and synonyms relating to cancer, exercise, implementation, facilitators, and healthcare settings and were created initially by GLW with subsequent input from two co-authors (GLR, MAK). The search strategy was then consulted with a qualified library liaison in health sciences (Supplementary Table 2). Terms were combined using Boolean operators, with truncation used where possible. Due to the general nature of the included terms, and to capture implementation focussed papers, the search strategy was limited to terms included in title and/or abstract. There were no date restrictions, nor any filters applied to ensure returns were not limited due to incomplete database indexing.

#### Selection criteria

The inclusion and exclusion criteria for this review are summarised in Supplementary Table 3. Briefly, studies were included if they met the following criteria: (i) people involved in the implementation process of an exercise service under “real-world” conditions; (ii) studies that explored the facilitators of implementing an exercise service pre-, during and/or -post treatment for adult people living with or beyond cancer (≥18 years); (iii) co-located exercise services or referral pathways (to external exercise services) within a cancer centre. Articles that involved paediatric populations, not real-world settings (e.g., clinical trials), not within a cancer centre setting, or where full text was not available and not provided after contact, were excluded. Implementation efforts that solely focused on exercise counselling/discussions without implementing an exercise service or referral pathway were excluded.

#### Article screening

All articles underwent automatic de-duplication (Covidence, Veritas Health, Australia) followed by manual de-duplication. Title/abstract and subsequent full text screening was conducted by at least two independent reviewers (GLW, BJC, RD, JM, CWG, MW) on the Covidence platform (Covidence, Veritas Health, Australia). Disagreements were resolved by discussion; where consensus was not reached, a third arbiter determined the final decision (GLR). Backwards and forwards citation chaining was used to identify additional eligible studies, namely the reference lists and citing studies of included studies, respectively.

#### Data extraction

Data extraction was completed by one author (GLW) using a pre-defined, electronic datasheet including author, country, stakeholders, setting, implementation effort, exercise service description, cancer continuum timepoint, and verbatim information regarding exercise implementation facilitators (and quotes where provided). If additional information was required that was pertinent to analysis (e.g., facilitator descriptions), the authors of the study were contacted. Facilitators reported by community-based deliverers (e.g., exercise professionals) external to the cancer centre (i.e., where cancer centres referred to an external service) were not extracted if the facilitator did not pertain to the operation of the referral pathway within the cancer centre, as this review focuses on the implementation within the context of cancer centres/hospitals. GLR conducted a 10% compliance check of extracted study details and facilitators. Discrepancies were 0% and 11% for study details and facilitators, respectively, and were resolved through discussion between both authors.

#### Data coding

Facilitators were deductively coded to the updated Consolidated Framework for Implementation Science (CFIR 2.0) [16] collaboratively by two authors (GLW and GLR). GLW has prior experience applying CFIR 2.0 in a study on the implementation of voluntary assisted dying discussions for general practitioners (Stroil-Salama et al*., in press, Aust J Gen Pract*), and GLR has experience applying CFIR 2.0 in ongoing research projects. The CFIR 2.0 guides the assessment and categorisation of barriers and facilitators across five domains — innovation, outer setting, inner setting, individuals, and implementation process [16]*.* The CFIR 2.0 framework was fully operationalised prior to coding, with all domains and constructs defined a priori using the published CFIR 2.0 definitions*. *Table 2 presents the CFIR 2.0 domains used in this review, with full construct descriptions available in Damschroder et al. — specifically in additional file 6 [16]. In the instance a facilitator could be coded across multiple domains or constructs, the facilitator was allocated to the CFIR 2.0 domain and construct that reflected the greatest impact as determined by GLW.

**Table 2:** Consolidated framework for implementation research 2.0 domains adapted from Damschroder et al. [16]

CFIR 2.0 domain	Definition [16]	Definition operationalised in this review
Innovation	The “thing” being implemented, e.g., a new clinical treatment, educational program, or city service	The exercise service being implemented
Outer setting	The setting in which the Inner Setting exists, e.g., hospital system, state. There may be multiple Outer Settings and/or multiple levels within the Outer Setting (e.g., community, system, state)	The broader context outside the cancer centre/hospital that influences implementation, such as funding systems, policies, and larger health system structures
Inner setting	The setting in which the innovation is implemented, e.g., hospital, city. There may be multiple Inner Settings and/or multiple levels within the Inner Setting, e.g., unit, team	The cancer centre/hospital in which implementation occurs
Individuals	The roles and characteristics of individuals that have influence and authority over implementation outcomes and are commonly involved in the implementation and/or delivery [24]	Roles and characteristics of individuals that have influence and authority of the implementation and/or delivery of the exercise service (e.g., clinicians, exercise professionals, executives, people living with or beyond cancer [only if served on the implementation team]) This can also include these individual’s perceptions of recipients (e.g., people living with or beyond cancer’ characteristics and needs) and how these perceptions helped complete implementation/delivery [24]
Implementation process	The activities and strategies used to implement the innovation	The activities and strategies used to implement the exercise service

#### Data synthesis

As this systematic review includes quantitative, qualitative, and mixed-methods study designs, and the output is qualitative, a narrative synthesis was performed [27]. Following coding, facilitators were grouped by CFIR 2.0 domains (main themes) and constructs (sub-themes). For each domain, the number and percentage of facilitators were reported, and for each construct the number of facilitators was reported. Within each construct, facilitators were described narratively and verbatim quotes from participants of the included studies were provided to contextualise the findings.

## Results

### Search and selection of studies

**Fig. 1:**
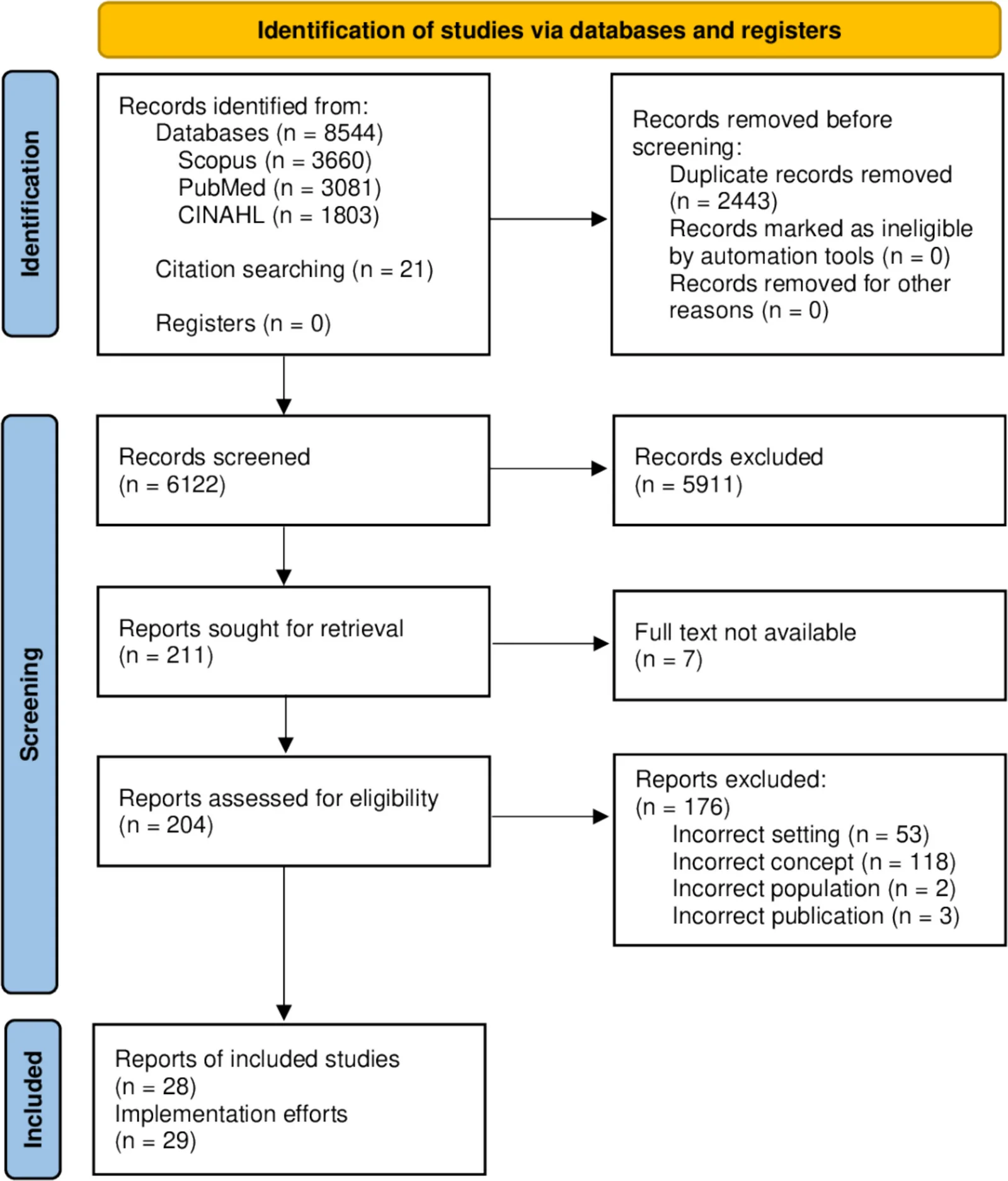
PRISMA diagram

As displayed in Fig. 1*,* the systematic search resulted in 8544 records. Following duplicate removal, the titles and abstracts of 6122 records were screened, with subsequent assessment of 211 full texts. A total of 28 reports were included, describing 29 implementation efforts [19–21, 28–52]. Both Czosnek and Santa Mina et al. reports included multiple implementation efforts [19, 31]. Multiple reports described the same implementation effort for four implementation efforts [21, 28–30, 32].

### Study and participant characteristics

Study and participant characteristics are shown in Supplementary Table 4 and 5. Most reports originated from either Australia (*n* = 9, 32%) or the United States of America (*n* = 8, 29%). Nearly all reports (*n* = 26, 93%) were pre-experimental (i.e., descriptive, cross-sectional, single group before and after, cohort) and only two reports were experimental (*n* = 2, 7%), specifically a randomised control trial [45] and type 1 hybrid effectiveness-implementation trial [28]. Just under half of the reports’ (*n* = 13, 46%) aims or methods related to the identification or assessment of implementation facilitators. Half of the reports employed mixed methods (*n* = 14, 50%), where the remaining were either qualitative (*n* = 10, 36%) or quantitative (*n* = 4, 14%). A total of 270 stakeholders from five stakeholder groups were represented across the studies and included referrers (i.e., oncologists, radiation oncologists, haematologists, nurses, surgeons, allied health), deliverers (exercise professionals e.g., exercise physiologists, physiotherapists), executives, researchers, and people living with or beyond cancer. A third of reports (*n* = 9, 32%) included stakeholders from multiple groups. Eleven reports (39%) did not include any stakeholders due to study design [21, 31–34, 36, 44, 49, 50, 52] or the exclusion of people living with or beyond cancer who were not directly involved in the implementation effort (as per this review’s exclusion criteria) [30]. Referrers represented more than half of all stakeholders (*n* = 161, 60%) and third of all stakeholders (*n* = 92, 34%) were exercise service deliverers. There were limited representatives from organisational (*n* = 9, 3%) [19, 20, 38], research (*n* = 7, 3%) [20], or people living with or beyond cancer that were directly involved in the implementation effort (*n* = 1, <1%) [20].

### Implementation effort contexts

Implementation efforts are described in Supplementary Table 4. Most exercise services were co-located (*n* = 16, 55%), followed by a combination of both co-located and referral pathways to external exercise services (e.g., multiple exercise services were offered) (*n* = 6, 21%), referral to solely external exercise services (*n* = 4, 11%), virtual (virtually co-located [*n* = 2, 7%], not reported if external or co-located [*n* = 1]) or not reported (*n* = 1, 3%). Exercise services were most frequently offered across the cancer continuum (*n* = 12, 41%), with some during-treatment (*n* = 8, 28%), post-treatment (*n* = 4, 14%), pre-treatment (*n* = 1, 4%), or not reported (*n* = 4, 14%). Most services included mixed cancer types (*n* = 20, 69%), with some instances of singular tumour streams (breast [*n* = 2, 7%], prostate [*n* = 1, 3%], colorectal [*n* = 1, 3%]).

### Facilitators to exercise oncology service implementation

**Fig. 2:**
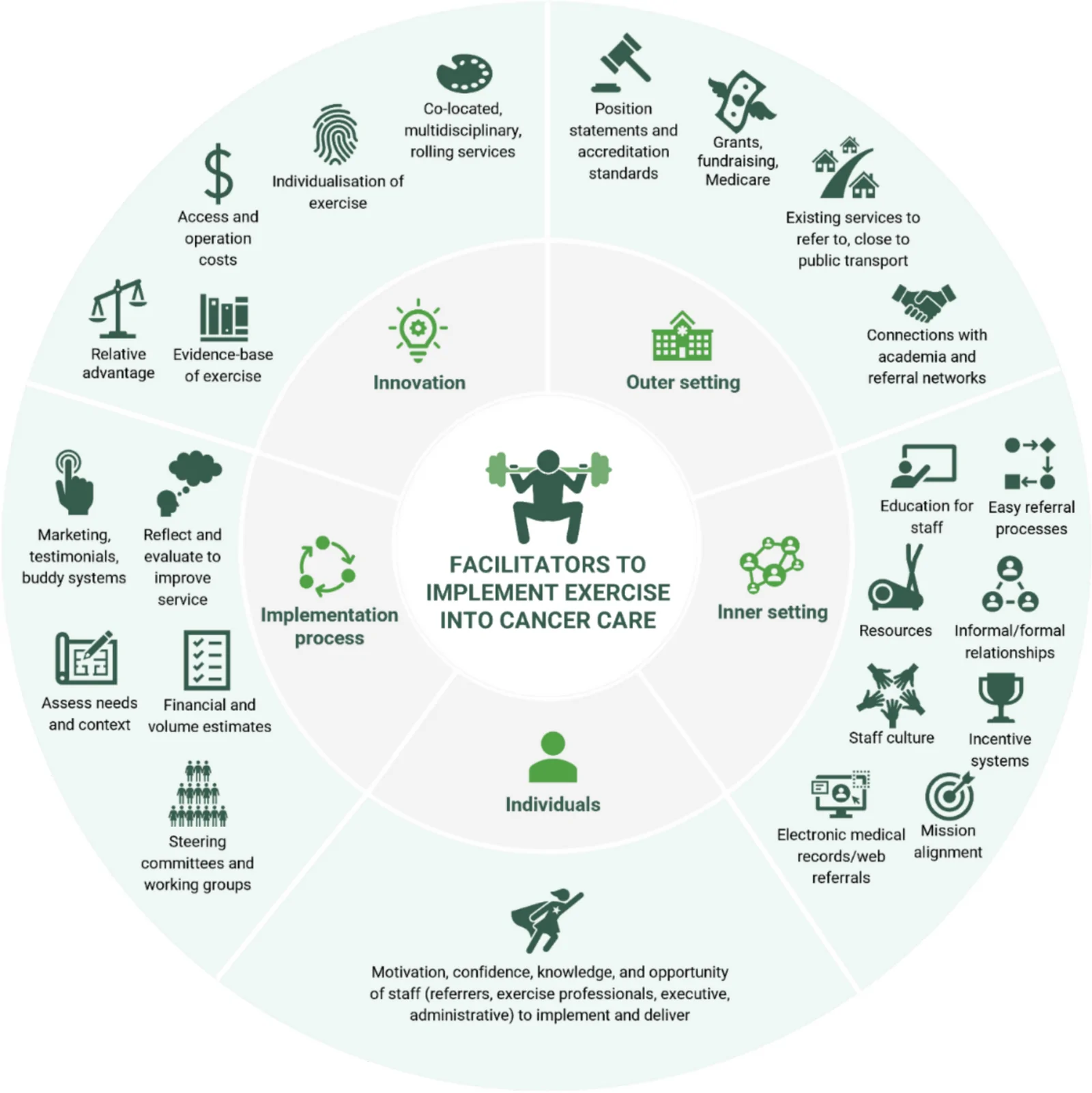
Facilitators to implement exercise into cancer care

A total of 164 facilitators were identified from 29 implementation efforts [19–21, 28–52] (Supplementary Table 6). Facilitators were identified across all five CFIR 2.0 domains; (i) inner setting (*n* = 49, 30%), (ii) implementation process (*n* = 45, 27%), (iii) innovation (*n* = 35, 21%), (iv) individuals (*n* = 24, 15%), and (iv) outer setting (*n* = 11, 7%). Figure 2 provides a visual overview of the summarised facilitators.

On average, there were six facilitators identified per implementation effort, across an average of three CFIR 2.0 domains (Supplementary Table 6). Across implementation efforts, several common co-occurring CFIR 2.0 constructs were identified. Pairs included: (i) access to knowledge with individual motivation (*n* = 8, 28%), (ii) access to knowledge and information with reflecting and evaluating (*n* = 8, 28%), and (iii) access to knowledge with compatibility (*n* = 7, 24%). Triplets included: (i) access to knowledge and information, individual motivation, reflecting and evaluating (*n* = 5, 17%), (ii) access to knowledge, compatibility, and teaming (*n* = 4 4, 14%), and (iii), access to knowledge, compatibility, and reflecting and evaluating (*n* = 4, 14%).

When reports in which the identification of facilitators was not the aim or included within the methods were removed from analysis (i.e., stated in the discussion, *n* = 15, 54%), the CFIR 2.0 domain order remained unchanged (inner setting *n* = 28 [30%], implementation process *n* = 25 [27%], innovation *n* = 22 [23%)] individuals *n* = 12 [13%], outer setting *n* = 7 [7%]).

#### Inner setting

Forty-nine inner-setting facilitators were identified from 18 implementation efforts [19–21, 28–32, 36, 38, 40, 43, 44, 48–52] and accounted for nearly a third of all facilitators (30%). Inner setting facilitators represented eight CFIR 2.0 constructs: (i) access to knowledge and information, (ii) compatibility, (iii) structural characteristics (information technology infrastructure), (iv), resources, (v) relational connections, (vi) culture, (vii) incentive systems, and (viii) mission alignment.

##### (i) Access to knowledge and information

Twenty facilitators were highlighted within this construct. Education regarding exercise oncology guidelines, evidence for exercise and cancer, as well as details of the local exercise service (implementation plans, processes, and purpose) for cancer care clinicians (e.g., medical oncologists, surgeons, nurses, allied health), exercise professionals, executive, and administrative teams was common. This included either generic or service-specific educational sessions/training [19, 20, 30, 31, 36, 48, 49, 52], presentations at grand-rounds and leadership meetings [31, 32, 50, 52], brochures, handouts, or scientific papers [20, 21, 28, 43, 50].

##### (ii) Compatibility, (iii) structural characteristics, (iv) resources

Compatibility of the innovation within workflow processes and systems represented 10 facilitators. Most of these facilitators related to the ease of referral processes. Strategies varied, however facilitator mechanisms included opt-out approaches [19], during chemotherapy infusion visits [50], and the option to choose from a range of referral methods that was most convenient for each referrer (i.e., in-person, or email, or phone call) [19, 29, 30]. A referrer (social worker) described the advantage of this flexibility and noted her preferred referral method bypassed the need for additional paperwork and saved time, “It was just an email, so very easy. ‘Cos it does irritate me, [other programs], you gotta fill in all this information… it took me 5–10 min instead of half an hour” [29]. Being provided pre-designed note templates for exercise consultations from a nationwide implementation network was also seen as helpful instead of needing to create these from scratch [51].

Structural characteristics, in particular, information technology infrastructure, was also a frequent construct (*n* = 10). Specifically, this related to the integration of electronic medical records or electronic web referrals [19, 20, 30–32, 40, 44, 50]. Two implementation efforts explained the integration of the referral process into the electronic medical record allowed direct communication from referrers to exercise professionals to initiate the referral, inform the reason for referral, medical history, contraindications and to monitor patient progress [31, 40]. It also increased exercise referrals and service expansion within one implementation effort [31]. Additionally, resources within cancer centres (*n* = 3) included the use of already existing exercise staff [51], gym equipment [30] and telehealth software [38] to assist in the implementation process.

##### (v) Relational connections, (vi) culture, (vii) incentive systems, (viii) mission alignment

Informal and formal relationships within cancer centres was described three times. Personal relationships between referrers (e.g., medical oncologists and nurses) and exercise professionals were seen to enable trust in the service and optimised referrals [19, 29]. One exercise professional underpinned this notion, “There is a lot of communication and that is a big strength of (site name), we have always been like a big family here. I sit in the same room as other oncologists, and they can see the work I am doing and the meetings I am having with members of the team” [19]. This notion was resounded by nurses, where both clinical and soft skills of exercise deliverers were seen as central “They need special clinicians…I don’t think it can just be run by any physio, and we’ve certainly seen that on the ward when we’ve had our physios rotate through… I think the right clinicians are really important to the success of the program” [29].

Furthering this, staff culture of resilience, comfort, and readiness to make organisational changes to implement the exercise service and make exercise standard care was important (*n* = 1) [43]. Interestingly, an incentive was highlighted as a facilitator in one implementation effort; the exercise service had previously won a prestigious award and this was seen by the staff as a way to support and leverage implementation [19]. A less frequent facilitator (*n* = 1) was to ensure the exercise service’s goals were “assessed (and updated as needed) to align with the mission of the oncology clinic” [52].

#### Implementation process

Facilitators relating to the implementation process were second most common; 45 facilitators (27%) were described in 21 implementation efforts [19–21, 28–33, 35–37, 39–41, 43, 45, 47, 49–52]. Implementation process facilitators were identified across six CFIR 2.0 constructs: (i) engaging recipients and deliverers, (ii) reflecting and evaluating, (iii) teaming, (iv) assessing needs, (v) assessing context, and (vi) planning.

##### (i) Engaging recipients and deliverers

Purposively engaging people living with or beyond cancer and/or referrers to participate in the innovation or implementation, respectively, was most common and was expressed 17 times. Marketing materials regarding exercise oncology benefits and/or the service to people living with or beyond cancer and clinicians were common, and included leaflets, brochures, newsletters, posters, and patient testimonials [29, 30, 37, 40, 45, 51, 52]. The provision of a formal referral, in conjunction with these resources, was described as vital by referrers [35, 40], “If we just give them the information (without an active referral), it’s, you know, it’s probably just gonna go in a paper file somewhere” [40]. Having exercise deliverers make initial contact with people living with or beyond cancer to either advise they would benefit from the exercise service (after multidisciplinary meetings) or to follow up after having received a referral, rather than people living with or beyond cancer having to do this of their own accord, was well received [21, 28, 35, 36]. Indeed, in one implementation effort, initial assessments increased from 39 to 65% having used this strategy [21, 28]. Clinicians highlighted the importance of referrer involvement to engage people living with or beyond cancer, particularly describing the benefits of exercise to their patients at the time of referral [47] or using peer involvement and “buddy systems” [37, 41, 47]. “We need to ‘sell’ the benefits of the program to the patients at the time of referral. In the same way they won’t take medications if they don’t realise the reason/importance of taking it” (referrer) [47]*.*

##### (ii) Reflecting and evaluating

Purposively reflecting on and evaluating the innovation and/or implementation was reported 12 times. Varying elements were evaluated, including service utilisation (number of referrals, new patient appointments) [19, 20, 31, 32, 52], innovation effectiveness (e.g., physical health outcomes) [31–33, 43, 49], barriers during implementation [43], effectiveness of referral processes [21, 28], financial sustainability [33] as well as revenue [20, 52] and costs of exercise deliverers [52]. Not only were these aspects evaluated, but it was common to use these outcomes to change and optimise service delivery and implementation processes, specifically to assist with referrals [21, 28] and inform allocation of resources to the executive team [19]. A staff member highlights the importance of regular evaluation, “We do track the effectiveness of what we're doing…. at the end of every week, I’m tracking how many new inquiries we had. How many of those people have transitioned to being clients? And if they’ve declined –why? Is it too expensive? Do they live too far away? So trying to capture the reasons why people aren’t taking part and then we can use that data to address things.” [19]. Three implementation efforts reported reflection and evaluation occurred during regular “check-ins” or meetings with implementation team members [20, 40, 50].

##### (iii) Teaming

The intentional collaboration or teaming between stakeholders (clinicians, researchers, implementation advisors, people living with or beyond cancer) was described eight times. The establishment of formal steering committees and working groups to drive the implementation effort was the most frequent way teaming was accomplished [19–21, 30–32, 50]. Most working groups or steering committees (*n* = 4, 67%) included people living with or beyond cancer [19–21, 30].

##### (iv) Assessing needs, (v) assessing context, (vi) planning

Collecting information about the need, priorities, and preferences of people, as well as barriers and facilitators to implementation (context), were also important and noted four and three times, respectively. For example, analysis and presentation of key information to the treating team (e.g., patient-reported outcome measures denoting extensive mobility impairment, frequent patient-requested services, patient satisfaction) initiated exercise service implementation across three efforts [19, 33, 52]. Perceptions of staff (e.g., nurses, psychologists) regarding the needs of their patients was likewise important to initiate exercise service inclusion [39]. Alongside the assessment of actual and perceived needs of patients to drive exercise service implementation, the regular review and management of staff readiness and engagement to partake in the implementation maintained service productivity even during leadership and staff turnover [21]. This was conducted through ongoing meetings with staff which increased the research team’s knowledge and understanding of the local context [21]. A less frequent facilitator was planning measures for implementation success in advance (*n* = 1), specifically developing a financial projection and patient volume estimate of the exercise service [52].

#### Innovation

Thirty-five (21%) innovation facilitators were described across 18 implementation efforts [19–21, 28–30, 35–41, 45, 46, 48, 49, 52]. Innovation facilitators were identified across five CFIR 2.0 constructs: (i) innovation adaptability, (ii) innovation evidence-base, (iii) innovation cost, (iv) innovation design, and (v) innovation relative advantage.

##### (i) Adaptability

The exercise service being adaptable to the setting’s needs or context was highlighted 13 times as an important factor. Specifically, the individualisation of exercise itself, considering and catering to all cancer continuum time points, patient goals and preferences, treatment history, cancer type/stage, and co-morbidities was seen as vital [19, 21, 28, 34, 35, 37, 40, 49]. “Cancer never comes by itself. There’s always multifaceted avenues and co-morbidities so it’s not just about the cancer when prescribing exercise” (exercise deliverer) [37].

##### (ii) Evidence-base, (iii) cost, (iv) design, (v) relative advantage

The evidence-base of exercise oncology was mentioned nine times and included differing sources of evidence that increased support for and referral to the exercise service. Sources included guidelines [29], documented benefits of exercise within the literature [19, 39, 45, 46], as well as referrer’s experiences of having seen or heard their patients benefit from the exercise service [29, 37, 52]. Costs to use or operate the exercise service was described six times. Services either employed a no or low-cost approach for patients to access the service [20, 29, 30, 38, 48] or parking fees [37]. Costs to operate the service were lower than previous in-person models of care [29, 30].

The design of exercise services was noted six times. Services that had a rolling program rather than block (patients starting and finishing the program at the same time) [37], appointments/classes on most weekdays [29, 30], co-located [29], or were integrated within a wider multidisciplinary service were seen as important facilitators [19]. These design aspects reinforced to referrers that exercise itself was important and safe (co-location) [29] and decreased cognitive load to refer (within a wider service) [19]. “…The success of our programme has really been taking a much more macro approach to exercise, and embedding that within the core delivery of our program… (by contrast) if you have an add-on service then it’s very, very hard for us to know who to refer to, and which patients we select to get it” [19]. Additionally, clinicians expressed a relative advantage (*n* = 1) of the implemented exercise service, stating it is better than another existing service within the hospital due to the latter’s “rigid structure and waiting times” [29, 30].

#### Individuals

Twenty-four facilitators (15%) from 18 implementation efforts [19–21, 28–31, 33, 34, 37, 38, 42, 43, 47, 48, 51, 52] were identified. Individual facilitators were categorised within one CFIR 2.0 construct, (i) innovation deliverers (people directly involved in the implementation process).

##### (i) Innovation deliverers

The motivation (*n* = 19), capability (knowledge) (*n* = 4), and opportunity (*n* = 1) mainly of referrers but also exercise professionals, executive, administrative and research staff were identified as facilitators. Motivation, specifically, championing behaviour, emerged as a key facilitator. “The engine was, I would still say, the great engagement and fire of single individuals that believe in this concept or stand for it” [51]*.* Specifically, nurses and oncologists valued and believed in exercise, which led to frequent referrals [37, 42, 43, 47, 48, 51]. Referrers also enjoyed being able to offer the exercise service for their patients as a “positive” experience for the person after a cancer diagnosis [48]. Additionally, championing behaviours of clinician, executive, administrative, and research staff leveraged resources [31, 52] and grants [50], limited project interruptions during staff and leadership turnovers [21] and established intersectoral relationships and communication [20, 21, 28, 34].

Importantly, the referrers’ capability, specifically knowledge and confidence, to speak to their patients about exercise was noted [19, 29, 30, 39] (*n* = 4). The opportunity to discuss or refer was a less frequent identified facilitator (*n* = 1). Referrers felt their skills and knowledge of exercise oncology was limited, and thus having an experienced exercise professional to refer to, instead of having to provide this exercise guidance themselves, was beneficial [39].

#### Outer setting

The least frequent domain (*n* = 11, 7%) identified facilitators from eight implementation efforts [19, 20, 29, 30, 34, 49, 51, 52]. Outer setting facilitators were described across four CFIR 2.0 constructs: (i) policies and law, (ii) financing, (iii) partnerships and connections, and (iv) local conditions.

##### (i) Policies and law

Professional group guidelines and recommendations, or accreditation standards that supported innovation delivery and implementation were mentioned four times. Specifically, Commission on Cancer (CoC), Clinical Oncology Society of Australia (COSA) exercise position statement [19], state cancer plans and reporting requirements [52] were seen to legitimise and leverage the exercise service.

##### (ii) Financing, (iii) partnerships and connections, (iv) local conditions

Full or partial funding (*n* = 3) of exercise services from external entities were secured by grants [29, 30, 49], Medicare Chronic Disease Management Plans [20], philanthropic funds [29, 30], or fundraising [49]. Partnerships and connections with external entities such as referral networks and academic affiliations were described twice. The establishment of internal and external referral pathways to cater for varying physical needs lead to an online network map of available exercise services, was instrumental [34]. One cancer organisation fostered relationships with universities to test and trial exercise service expansions [19]. Local conditions (*n* = 2 facilitators, *n* = 2 implementation efforts) such as the location of the exercise service being close to public transport and oncology clinics [49], or existing exercise services available that oncology clinics to refer to [51], were seen to support implementation.

## Discussion

This review has comprehensively collated 164 facilitators that assist the implementation of exercise oncology services within real-world settings. Facilitators were identified across all five CFIR 2.0 domains which indicates buy-in and engagement are important across multiple healthcare levels. Further, including a variety of stakeholders is crucial. The most common facilitators within this review were categorised within the *inner setting* (30%) and *implementation process* (27%) domains. Therefore, exploring and understanding opportunities at these levels might serve as a helpful starting point for implementation efforts. Interestingly, barriers in exercise oncology implementation have previously been identified across multiple healthcare levels, also with a key focus on the organisation [18]. Separately, Czosnek et al. synthesised implementation outcomes of exercise oncology programs [22]. Both implementation outcomes (the “what”) and determinants (the “how” — facilitators and barriers) are important for understanding exercise oncology implementation. Together, these previous reviews by Kennedy et al. [18] and Czosnek et al. [22], alongside this review, provide a comprehensive picture of exercise oncology implementation. Both outcomes and influencing factors can guide the design and implementation of future exercise oncology services within cancer care settings.

The most common CFIR 2.0 constructs that aided implementation success were (i) access to knowledge and information among the referring and delivering team, (ii) engaging people living with or beyond cancer and referrers, (iii) motivation (particularly of referrers), (iv) adaptability of the innovation and (v) reflecting and evaluating on implementation and innovation success. This review suggests these facilitators were commonly present across implementation efforts and may represent foundational factors to consider in implementation planning. However, any given facilitator will always depend on how well it aligns with the specific setting, resources, and stakeholder needs. Therefore, even global facilitators need to be adapted to each unique implementation effort and context [17]. For example, the facilitator of electronic medical records [19, 20, 30–32, 44] within the *structural characteristics* construct may not be a feasible enabler for a cancer centre or hospital that rely on paper-based systems. Similarly, the *financing* facilitator of asking patients to access a Medicare Chronic Disease Management Plans to offset the operational costs of the exercise service [20], is not transferrable in countries other than Australia.

Implementation facilitators rarely act in isolation, rather an interconnected network of facilitators work together to bridge the research to practice gap in exercise oncology settings. Implementation efforts in this review included an average of six facilitators, coded across an average of three CFIR 2.0 domains. Clusters of the most frequent CFIR 2.0 constructs also underpin this interaction. For example, characteristics of *individuals* (directly involved in delivering the innovation e.g., referrers, exercise professionals) including motivation, can be fostered by facilitators in other CFIR 2.0 domains such as having *access to knowledge and information (inner setting)* and having *trust and confidence (inner setting: relational connections)* of exercise professionals. This interaction may indicate that even though individual championing behaviour can be heavily relied upon and seems to be a driving force in an implementation effort, it does not operate in isolation. Organisational context can influence such behaviour which is underpinned by the fact most facilitators existed within the *inner setting*. Facilitators in the *implementation process,* such as *assessing context and needs, reflecting and evaluating,* as well as *teaming,* can influence *innovation adaptability (innovation)* and *workflow compatibility (inner setting).* These *implementation process* constructs shape how the exercise service fits into the specific context and workflows of the inner setting. Identifying and acting upon facilitators and barriers pre- and during implementation, especially with stakeholder groups, ensures the exercise service is context-specific, stands the best chance possible, and evolves with its environment [53, 54]. Importantly, *adaptability* was the most frequent construct within the *innovation* domain which underpins the importance of individualisation of exercise for people living with or beyond cancer, as recommended by exercise guidelines and position statements [8, 11, 12].

Facilitators in the *outer setting* were less frequent (6%), despite playing an important role in implementation and sustainability of healthcare services [55]. Alignment with *policies and law,* specifically the Commission on Cancer (CoC) and Clinical Oncology Society of Australia (COSA) exercise position statement, were seen to legitimise *innovation evidence-base* [19, 52], and may foster *relational connections (inner setting), partnerships and connections (outer setting), as well as motivation of individuals (individuals).* Within this review, only three funding facilitators were identified [20, 29, 30, 49] and *innovation cost* facilitators were commonly related to patient costs, rather than organisational costs. The majority of barriers to exercise oncology implementation have been described at the organisational level, which includes issues such as funding [18]. Cost outcomes including funding models, are infrequently conducted among exercise oncology implementation efforts [22]. The minimal frequency of *outer setting* facilitators may indicate a true lack of enablers within implementation efforts, or under evaluating/reporting, as well as a lack of relevant stakeholders, such as organisational representative perspectives.

Perspectives from organisation representatives or people living with or beyond cancer (that were directly involved in the implementation effort) comprised less than 4% of total stakeholders. Most stakeholders were either referrers (*n* = 161, 60%) or exercise deliverers (*n* = 92, 34%). Furthering this, there were only four implementation efforts (14%) that included people living with or beyond cancer on implementation teams or as key-decision makers [19–21, 29, 30]. Implementation success, as well as service access and effectiveness, is only possible when there is equitable power and joint decision-making across all key [56]. As the exercise oncology implementation field progresses, it will be important to ensure multiple stakeholders, especially people living with or beyond cancer that have influence over implementation outcomes as well as organisational representatives, are meaningfully involved in efforts.

Although not the focus of this review, limited implementation efforts (*n* = 6, 21%) prospectively or retrospectively identified implementation strategies, specifically from the Expert Recommendations for Implementing Change (ERIC) taxonomy [57]. The ERIC taxonomy was published in 2015 (all included reports, except one, within this review were published after 2015). The taxonomy provides 73 practical implementation strategies, and each can be mapped to the relevant CFIR 2.0 domain, that can be selected and designed when building an implementation plan [57]. To progress exercise oncology implementation and inform future efforts, the utilisation and documentation of ERIC strategies is encouraged to enhance generalisability and foster a shared language within the field.

### Practical implications

Without understanding implementation barriers and facilitators, implementation efforts will rely on one-size-fits-all or trial-and-error approaches, which often limit success. Identifying and leveraging facilitators can help organisations move beyond trial-and-error and design implementation efforts that are more likely to succeed. For organisations aiming to bridge the research-to-practice gap in exercise oncology, facilitators in the *inner setting* (staff education, streamlined referrals, strong relationships) and *implementation process* (steering committees, stakeholder engagement, planning, reflecting) may serve as a strong starting point when designing their own implementation plans. However, facilitators are context-specific and their relevance depends on resources, needs, and priorities.

Consulting existing evidence, including barriers summarised by Kennedy et al. [18] and facilitators from this review, can help stakeholders anticipate challenges and identify opportunities for successful exercise oncology implementation. This insight can then be combined with a local needs assessment to identify facilitators and barriers within stakeholders’ own context. Using both global and context-specific determinants allows organisations to develop tailored, relevant, and practical implementation plans and services that stand the best chance possible.

## Limitations

This review is not without limitations. Certain search terms were purposefully removed from the search strategy, such as rehabilitation, refer, and discuss, due to a large number of search returns (>20,000). Therefore, it is possible reports that used these terms might not be included. This review also focused on exercise service provision, rather than solely exercise counselling or discussions. However, implementation efforts that included counselling or discussions as well as service implementation were included. It was common that the identification of facilitators was not the aim or included within the methods of the included reports and thus facilitators were sometimes reported within the discussion. However, when these reports were removed, the CFIR 2.0 domain order remained unchanged. Additionally, over a third of reports did not include stakeholder perspectives. This makes it difficult to understand facilitators from cancer care clinician, organisational, and people living with or beyond cancer representatives.

It is important to highlight this review utilised the CFIR 2.0 as the chosen implementation determinant framework. As noted within the methods section, CFIR 2.0 is not appropriate to use to collect recipient experiences unless they have authority and influence over implementation. The authors are aware many implementation science determinant frameworks exist which may indeed include recipient experiences as determinants (facilitators and/or barriers). We also acknowledge that, in real-world settings, cancer centres or hospitals may implement exercise services without publishing their experiences or findings. This gap between routine clinical practice and peer-reviewed publication may have limited the search results and findings. A traditional quality appraisal of individual studies was not conducted, as this review focused on synthesising facilitators rather than implementation outcomes. Instead, we assessed whether facilitators were explicitly documented within the study aims and methods, and studies in which facilitators did not fall into these categories were excluded; their removal did not change the CFIR 2.0 domain frequency results.

## Conclusion

Closing the exercise oncology research to practice gap is a major focus of the field; however, it is no easy feat; healthcare systems are dynamic and complex, influenced by numerous factors. Nearly a third of facilitators existed within the inner setting, which underpins the importance of an organisation’s capacity and willingness to prioritise, resource, and embed exercise for implementation efforts to stand the best chance possible. Despite this, it is important to realise successful implementation is not operationalised by a single driving factor but is instead supported by a range of interconnected facilitators. Summarising global facilitators provides actionable guidance for stakeholders, specifically oncology clinicians, exercise professionals, policy makers, and researchers, interested in pursuing and planning their own implementation efforts. The results of this review suggest it is the synergy of these global facilitators, anchored by an organisation’s resources and willingness, that propel exercise oncology implementation efforts and ensure people living with, or beyond cancer have access to exercise services.

## Supplementary Information

Below is the link to the electronic supplementary material.

ESM 1 (ZIP 150 KB)

## Data Availability

All data generated or analysed during this study are included in this published article [and its supplementary information files].
