# Supplementary material for: Facilitators to the implementation of exercise into cancer care: a systematic review
Source: J Cancer Surviv. 2026 Jan 4;20(Suppl 2):2237–50. doi: 10.1007/s11764-025-01948-5 (PMC13744452; doi:10.1007/s11764-025-01948-5)
Supplement: Supplementary file 1 [file 11764_2025_1948_MOESM1_ESM.zip › Supplementary/Supplementary Table 2.docx]

**Supplementary Table 2.** Search strategy

Facilitators to the implementation of exercise into cancer care: a systematic review. Journal of Cancer Survivorship. Georgia L. White, Mary A. Kennedy, Brent Cunningham, Riley Dunn, Jason Martin, Meegan Walker, Craig T. Wallington-Gates, Hattie Wright, Bryan Chan, Grace L. Rose

**Correspondence:**

Georgia L White

University of the Sunshine Coast, Queensland, Australia

E-mail: [georgia.white@research.usc.edu.au](mailto:georgia.white@research.usc.edu.au)

| #1 | cancer OR oncolog* |
| --- | --- |
| #2 | exercis* OR physical activit* |
| #3 | implement* OR integrat* OR strateg* OR facilitat* |
| #4 | healthcare OR health care OR hospital* OR centre* OR center* OR clinic* OR telehealth OR virtual OR community |
| #5 | **#1 AND #2 AND #3 AND #4** |

**PubMed**

("cancer"[Title/Abstract] OR "oncolog*"[Title/Abstract]) AND ("exercis*"[Title/Abstract] OR "physical activit*"[Title/Abstract]) AND ("implement*"[Title/Abstract] OR "integrat*"[Title/Abstract] OR "strateg*"[Title/Abstract] OR "facilitat*"[Title/Abstract]) AND ("healthcare"[Title/Abstract] OR "health care"[Title/Abstract] OR "hospital*"[Title/Abstract] OR "centre*"[Title/Abstract] OR "center*"[Title/Abstract] OR "clinic*"[Title/Abstract] OR "telehealth"[Title/Abstract] OR "virtual"[Title/Abstract] OR "community"[Title/Abstract])

**Scopus**

( TITLE-ABS-KEY ( cancer OR oncolog* ) ) AND ( TITLE-ABS-KEY ( exercis* OR physical activit* ) ) AND ( TITLE-ABS-KEY ( implement* OR integrat* OR strateg* OR facilitat* ) ) AND ( TITLE-ABS-KEY ( healthcare OR health care OR hospital* OR centre* OR center* OR clinic* OR telehealth OR virtual OR community ) )

**CINAHL**

XB (cancer OR oncolog*) AND XB (exercis* OR physical activit*) AND XB (implement* OR integrat* OR strateg* OR facilitat*) AND XB (healthcare OR health care OR hospital* OR centre* OR center* OR clinic* OR telehealth OR virtual OR community)
