# Supplementary material for: Facilitators to the implementation of exercise into cancer care: a systematic review
Source: J Cancer Surviv. 2026 Jan 4;20(Suppl 2):2237–50. doi: 10.1007/s11764-025-01948-5 (PMC13744452; doi:10.1007/s11764-025-01948-5)
Supplement: Supplementary file 1 [file 11764_2025_1948_MOESM1_ESM.zip › Supplementary/Supplementary Table 3.docx]

**Supplementary Table 3.** Inclusion and exclusion criteria

Facilitators to the implementation of exercise into cancer care: a systematic review. Journal of Cancer Survivorship. Georgia L. White, Mary A. Kennedy, Brent Cunningham, Riley Dunn, Jason Martin, Meegan Walker, Craig T. Wallington-Gates, Hattie Wright, Bryan Chan, Grace L. Rose

**Correspondence:**

Georgia L White

University of the Sunshine Coast, Queensland, Australia

E-mail: georgia.white@research.usc.edu.au

Inclusion criteria

- People involved in the implementation and/or delivery of an exercise service under “real world” conditions, including:
  - People receiving exercise oncology services only if they have been a key-decision maker (authority over the implementation/delivery):
    - Adults (≥18 years) living with or beyond cancer (from time of diagnosis to end of life)
  - People delivering/offering exercise oncology services:
    - Organisations (e.g., executives, policy makers)
    - Health professionals (e.g., exercise physiologists, physiotherapists, exercise professionals)
  - People referring to services:
    - Health/medical professionals (e.g., oncologists, surgeons, nurses, allied health professionals)

- Studies that explore the facilitators of implementing an exercise service pre, during and/or post-treatment for adults living with or beyond cancer.

- Cancer centre/hospital setting (private and/or public)
  - This refers to co-located exercise services or referral pathways (to physical or tele-delivered services) integrated within cancer centres (i.e., standalone cancer centres or cancer centres within a hospital)
  - Exercise service refers to a program or clinic designed to deliver exercise to people living with or beyond cancer
    - Exercise is defined as any form of planned, structured, and repetitive bodily movement performed to improve or maintain fitness or health

- Any quantitative (e.g., RCTs, quasi RCT, pre-post interventions, cohort studies)
- Any qualitative
- Mixed methods

Exclusion criteria

- Paediatric exercise service(s) (<18 years)

- People not involved in the implementation process (e.g., adults living with or beyond cancer not serving on an implementation team or effort)

- Proposed or recommended facilitators (not experienced facilitators)

- Facilitators unrelated to issues of implementation

- Facilitators of the exercise service cannot be isolated from other embedded intervention facilitators within the service (i.e., psychology, dietetics)

- Service is not within or referred from the cancer centre/hospital

- Manual therapy, not explicit exercise

- No full text available

- Incorrect publication type (e.g., poster presentation, conference proceedings, review article, protocol)

- Published in languages other than English that are unable to be electronically translated

- Studies unrelated to issues of implementation (e.g., clinical trials)
