# Supplementary material for: Facilitators to the implementation of exercise into cancer care: a systematic review
Source: J Cancer Surviv. 2026 Jan 4;20(Suppl 2):2237–50. doi: 10.1007/s11764-025-01948-5 (PMC13744452; doi:10.1007/s11764-025-01948-5)
Supplement: Supplementary file 1 [file 11764_2025_1948_MOESM1_ESM.zip › Supplementary/Supplementary Table 4.docx]

**Supplementary Table 4.** Overview of included reports by implementation effort

Facilitators to the implementation of exercise into cancer care: a systematic review. Journal of Cancer Survivorship. Georgia L. White, Mary A. Kennedy, Brent Cunningham, Riley Dunn, Jason Martin, Meegan Walker, Craig T. Wallington-Gates, Hattie Wright, Bryan Chan, Grace L. Rose

**Correspondence:**

Georgia L White

University of the Sunshine Coast, Queensland, Australia

E-mail: georgia.white@research.usc.edu.au

| Study | Aim | Study design | Study aim(s) or methods related to identifying facilitators | Methods | Implementation effort |
| --- | --- | --- | --- | --- | --- |
| Author  Year  Country |  |  |  |  | **S:** healthcare setting  **L:** service delivery location  **C:** application of cancer continuum timepoint  **T:** cancer type  **D**: exercise service/referral pathway description |
| Adsul et al., 2022  USA | **Case A**  Identify common and differentiating aspects of the implementation process and strategies used in three different implementation efforts focused on exercise evidence-based interventions. | Pre-experimental | Y | Qualitative | **S:** Rural community cancer care.  **L:** Referral to external service.  **C:** Post-treatment.  **T:** Mixed.  **D:** 12 week supervised in-person aerobic exercise sessions during the first 6 week tapered to fully independent, unsupervised aerobic exercise by the last 6 week. During the last 6 week, participants also met biweekly with exercise staff to provide updates on adherence and receive physical activity counselling and recommendations. Participants also attended six discussion group meetings throughout the physical activity program during which they received education and counselling targeting self-efficacy, exercise barriers, goal setting, time management. |
| Adsul et al., 2022  USA  Beidas et al., 2014  USA | **Case C**  Identify common and differentiating aspects of the implementation process and strategies used in three different implementation efforts focused on exercise evidence-based interventions.  Quantitatively assess whether an evidence-based exercise intervention for breast cancer survivors, Strength After Breast Cancer, was safe and effective in a new setting and to qualitatively assess barriers to implementation. | Pre-experimental  Experimental (hybrid effectiveness-implementation type 1) | Y  N  (only specified barriers) | Qualitative  Mixed methods | **S:** National Cancer Institute designated cancer centre  **L:** Co-located.  **C:** Post-treatment.  **T:** Breast.  **D:** Survivors referred to the program from oncology clinicians (physicians and nurse practitioners) and physical therapists at a National Cancer Institute designated comprehensive cancer centre and by self-referral. Survivors were eligible for referral into the program if they had completed all curative treatments (except for hormonal therapies and trastuzumab) and if the referring clinician did not find any medical conditions that would preclude participation. Lymphedema status had no bearing on eligibility for referral. 1 physical therapists to <7 survivors. 4x group sessions over 1-2 months, strength home exercise in between. |
| Barnes et al., 2020  USA | Describe the implementation of a clinically integrated physical therapist in oncologic care teams. | Pre-experimental | Y | Qualitative | **S:** Huntsman Cancer Institute in the University of Utah Health.  **L:** Co-located.  **C:** During-treatment.  **T:** Mixed.  **D:** A clinically integrated physical therapist within an oncology centre. The physical therapist uses a risk stratification tool to triage to the appropriate exercise modality. Home exercise prescription ranges from stage 1 (basic transfer and mobility exercises) to stage 5 (15 min resistance and 30 min aerobic exercises). |
| Brennan et al., 2024  Ireland | Evaluate the feasibility of the personalised exercise rehabilitation in cancer survivorship (PERCS) triage and referral system. | Pre-experimental | N | Qualitative | **S:** St James’s Hospital, Dublin.  **L:** Co-located.  **C:** Post-treatment.  **T:** Mixed.  **D:** PERCS Exercise Rehabilitation Triage and Referral System involves three steps:   1) Patient assessment: physical activity levels, past medical history, falls risk (Timed up and Go) and Eastern Cooperative Oncology Group score. 2) Triage: 2 questions    a. Is the person already meeting the recommended guidelines for exercise? (Aerobic exercise: at least 30 minutes moderate intensity, 3 times per week, and strength training: at least 30 minutes, 2 or more days per week)    b. Would this person be safe exercising without medical supervision?   Based on responses, participant is allocated to one of three recommended pathways:   a. Level 1: advice, continue with current exercise b. Level 2: refer to exercise with fitness professional c. Level 3: refer to exercise with rehab professional 3) Personalised physical activity plan: a discussion of triage outcome takes place with the patient, collaborative creation of an exercise plan and making any required referrals to exercise rehabilitation services.   Participants received a follow-up call 1 week from the triage physiotherapist to address any issues and facilitate progress with exercise goals. |
| Caperchione et al., 2023  Australia | Evaluate the feasibility and acceptability of an evidence-based approach to improving exercise communication between cancer care clinicians and their patients, inclusive of an exercise referral pathway from clinician to exercise physiologist. | Pre-experimental | N | Qualitative | **S:** Public hospital, Sydney.  **L:** Referral to external service.  **C:** Across the continuum.  **T:** Mixed.  **D:** Exercise communication and referral pathway approach. Cancer care clinicians employed the "Assess, Aware, Advise" pathway. Clinicians were to engage in a brief (1–2 min) conversation about exercise with their cancer patients during a regular consultation or appointment. If appropriate they referred patients to a designated EP. Patients were contacted by an EP via telephone. EP counselling lasted approximately 15–20 min and included: a brief medical and physical activity history, personalised physical activity recommendations based on patients’ disease stage/type, preferences and interests, identification of local EP clinics and other local exercise opportunities, and education regarding Medicare rebated EP sessions. |
| Czosnek et al., 2023  Australia | Systematically identify the determinants, implementation strategies (including mechanisms of change), and implementation outcomes for exercise EBIs in cancer care based on established implementation science frameworks. | Pre-experimental | Y | Mixed methods | **Case A**  **S:** Publicly funded healthcare facility in New South Wales.  **L:** Co-located.  **C:** During-treatment.  **T:** Mixed.  **D:** The survivorship service was established in 2013 and is accessible to anyone who undergoes active cancer treatment at the site. patients undergo an initial review with members of a multi-disciplinary team that includes an oncologist, nurse, psychologist, dietitian, and an EP The multi-disciplinary team then develops a holistic treatment plan.  12-week program (15 contact hours)   - 1x20 minute introduction + 1x1 hour initial assessment - 1x1hr/week for 12 weeks - 1x1 hour final assessment - 1x20 minute review at 4 and 6 months - Sessions include: - 20 minutes aerobic exercise - 30 minutes of resistance exercise (6-8 upper and lower body exercises) - 10 minutes (group) stretching |
|  |  |  |  |  | **Case C**  **S:** Publicly funded healthcare facility. main campus is in metropolitan Melbourne, with four satellite sites located in local neighbourhoods and regional areas across Victoria.  **L:** Co-located.  **C:** Pre-treatment.  **D:** Delivers adult cancer services through 13 cancer streams. the exercise service is situated within a multi-disciplinary prehabilitation program and as a standalone allied health service that inpatients and outpatients can access. The prehabilitation program includes a comprehensive assessment and an established care plan that contains interventions from various disciplines (i.e., psychology, dietetics, EP). The stand-alone allied health service typically offers limited sessions and supports on-referral to exercise opportunities in the community where possible.   - 1x30 minute initial assessment - Session duration varies between 20 mins - 1 hour duration (onsite) - Re-assessment as required - Onsite sessions include: - 20 minutes aerobic exercise - 30 minutes (8 upper and lower body resistance exercise (2 sets 10-12 repetitions) - 10 minutes stretching - (preference is given to aerobic exercise when time is limited) |
| Dalzell et al., 2017  Canada | NR | Pre-experimental | N | Qualitative | **S:** Segal Cancer Centre.  **L:** Co-located.  **C:** Across the continuum.  **T:** Mixed.  **D:** Patients have access to a well-equipped gymnasium and a broad variety of exercise classes staffed by volunteers and overseen by exercise specialists. Programs were based on recommendations provided by the American College of Sports Medicine roundtable on exercise guidelines for cancer survivors and included components of flexibility, cardiovascular, and resistance training whenever possible. Programs were personalised to include a combination of home exercise, Wellness Centre–based training, or participation in exercise classes, depending on the status of their disease and treatments at any given time. Patient re-assessments were scheduled every 3 months, at which time the kinesiologist discussed the patient’s compliance and barriers, re-assessed physical functioning, and established new goals. If unable to attend in person, recommendations were provided for use of the most accessible community-based exercise facilities available, and communication was established with trainers as needed. |
| Dennett et al., 2017  Australia | (1) Describe oncology rehabilitation programs provided in Australia with a focus on exercise therapy, (2) determine whether the exercise component of programs is consistent with guidelines, and (3) identify barriers and facilitators to oncology rehabilitation program implementation. | Pre-experimental | Y | Mixed methods | **S:** Public and private hospital and cancer centres across Australia.  **L:** Co-located.  **C:** Across the continuum.  **T:** Mixed.  **D:** Mainly in person group exercise classes, with individualised exercise dosage for each survivor, supervised by physiotherapists or EPs. Reported mixture of resistance, aerobic, and flexibility exercises within services. A variety of strategies were adopted to encourage this including written home exercise programs and referrals to community groups. |
| Dennett et al., 2021  Australia | Complete a process evaluation of an exercise-based cancer telerehabilitation program. | Pre-experimental | N | Mixed methods | **S:** Large publicly funded health network in metropolitan Melbourne.  **L:** Telehealth (co-located).  **C:** Across the continuum.  **T:** Mixed.  **D:** 8-week supervised program delivered by a nurse coordinator, 3 physiotherapists, and an allied health assistant. Individualised exercise program based on initial consultation and goals.   - 1x 1:1 weekly 30 min online health coaching - 1x weekly 45 min online group supervised training - 1x weekly 45 min online group education. |
| Dennett et al., 2021  Australia  Dennett et al., 2021  Australia | Explore the clinician experience of the feasibility of a supervised, exercise-based cancer rehabilitation program co-located within a cancer treatment unit  Determine the feasibility of implementing a flexible, exercise-based cancer rehabilitation program in a hospital-based cancer unit. | Pre-experimental  Pre-experimental | Y  N | Qualitative  Mixed methods | **S:** Publicly funded tertiary hospital in metropolitan Melbourne, Australia  **L:** Co-located.  **C:** Across the continuum.  **T:** Mixed.  **D:** 8-week supervised, rolling, exercise-based rehabilitation program, with a maximum of 8 participants per group. Participants completed a 1hr comprehensive assessment with a physiotherapist and were invited to attend an individually tailored, supervised group-based circuit exercise class once or twice weekly or be provided with a tailored home exercise program for 8 weeks. Participants opting for home-based training received an initial face-to-face assessment only and an 8-week follow-up in-person or via telephone. Exercise was prescribed in accordance with guidelines. |
| Devenney et al., 2023  Ireland | Examine the feasibility of implementing a 10-week multi-modal exercise rehabilitation programme into the management of deconditioned cancer survivors in a national cancer centre. | Pre-experimental | N | Mixed methods | **S:** St James’s Hospital, Dublin  **L:** Co-located.  **C:** Post-treatment.  **T:** Mixed.  **D:** 10-week multi-modal (combined aerobic, resistance, balance and flexibility) exercise program and includes twice weekly group exercise sessions. |
| Faro et al., 2023  USA | To develop and test ActivityChoice, an electronic referral (eReferral) clinic implementation program referring cancer survivors to physical activity programs of their choice. | Pre-experimental | Y | Mixed methods | **S:** Three sites affiliated with the Cancer Centre at UMass Memorial Health System in Worcester, Massachusetts: one academic medical centre (UMass Memorial Medical Centre), and two community hospitals (Marlborough Hospital and HealthAlliance-Clinton Hospital)  **L:** Referral to external service (YMCA) or telehealth (Fit Cancer)  **C:** Across the continuum.  **T:** Mixed.  **D:** ActivityChoice is a clinic-based eReferral program, with 5 steps:   1) clinician discusses choices of exercise programs 2) patient chooses program 3) clinician e refers patient 4) text and email confirmation of referral 5) program contacts and enrols patient   The two exercise programs offered:   1) LIVESTRONG at the YMCA program: a 12-week in-person program for cancer survivors led by qualified exercise professionals meeting 2×/week focusing on aerobic, strength, balance, and flexibility exercises. 2) Fit Cancer: 8-week virtual program meeting once per week for cancer survivors. |
| Ijsbrandy et al., 2020  Netherlands | Identify the barriers and facilitators affecting physical activity programme implementation delivering in a shared-care model. | Pre-experimental | Y | Qualitative | **S:** Hospitals (categorical, university, teaching, and non-teaching)  **L:** Referral to external service and co-located.  **C:** NR.  **T:** NR.  **D:** Physical activity programmes are offered by (1) rehabilitation physicians in hospitals or rehabilitation clinics; apart from physical problems, patients also need to have been diagnosed with psychological or social problems to be allowed to participate in these physical activity programmes; (2) multiple healthcare professionals (e.g. sports-medicine physicians, physiotherapists) or by non-healthcare professionals such as sports trainers; these programmes are mainly offered outside of the hospitals. The majority (> 70%) of patients only attends the second physical activity programmes. |
| Kennedy et al., 2020  Australia | Provide insight into the co-located exercise clinic operational success using the RE-AIM (Reach, Effectiveness, Adoption, Implementation, Maintenance) framework. | Pre-experimental | N | Mixed methods | **S:** GenesisCare clinic in Joondalup, Western Australia  **L:** Co-located.  **C:** Across the continuum.  **T:** Mixed.  **D:** An accredited exercise physiologist delivered the exercise programme. The EP offered individual assessments and oversaw group exercise sessions 2–3 days/week. |
| Kennedy et al., 2022  Australia | Evaluate the implementation outcomes of the co-located exercise clinic after operationalization of the implementation plan. | Pre-experimental | N  (only specified barriers) | Mixed methods | **S:** GenesisCare clinic in Joondalup, Western Australia  **L:** Co-located.  **C:** Across the continuum.  **T:** Mixed.  **D:** An accredited exercise physiologist delivered the exercise programme and spent one full day per week at clinic 1 (8 hr) and two full days per week at clinic 2 (16 hrs). Initial exercise assessments were conducted by the EP. |
| Kim et al., 2024  USA | Describe a collaborative process using a community-based provider participation in research to develop and test an implementation strategy (Exercise: Assess, Advise, Refer) to implement exercise is medicine into a community-based oncology clinic and measure associated intervention and implementation outcomes at baseline and post-implementation. | Pre-experimental | Y | Quantitative | **S:** Community oncology clinic in Washington State.  **L:** Telehealth (NR if co-located or external).  **C:** During treatment.  **T:** NR.  **D:** Assess patients attending the clinic for cancer treatment for their ability to exercise using PAVS/ECOG (Physical Activity Vital Signs/ Eastern Cooperative Oncology Group (ECOG) Performance Status) provide advice on exercise benefits; and refer 20 patients to a 3-month biweekly online exercise class, delivered by an American College of Sports Medicine-certified exercise trainer, with weekly monitoring of frequency/intensity of exercise and physical functioning using PAVS/ECOG. |
| Leach et al., 2018  USA | Describe the implementation of a physical therapy–based exercise program delivered within a health care setting for patients who have had cancer and (2) evaluate its effectiveness for reducing fatigue and improving aerobic capacity. | Pre-experimental | N | Quantitative | **S: "**Healthcare setting"  **L:** Co-located.  **C:** Across the continuum.  **T:** Mixed.  **D:** Patients received an initial evaluation and treatment visit with a physical therapist who developed the individualized exercise program for each patient. In the first evaluation session, the physical therapist identified any functional deficits (i.e., postmastectomy care, lymphedema management, pelvic floor dysfunction, swallowing deficits, cognitive deficits, vestibular deficits, etc) and, if indicated, recommended additional skilled physical therapy sessions. Following the first 2 visits with the physical therapist, patients were referred to the exercise program. These patients then paid $6 per session to exercise twice per week under the supervision of a Clinical Cancer Exercise Specialist (CCES; https://www.cancerrehabexercise.com). After approximately 3 months, a re-evaluation was scheduled with the same physical therapist.  Sessions lasted approximately 1 hr and consisted of low- to moderate-intensity aerobic, resistance, and balance exercises. Exercise intensity, duration, and modality were determined for each patient by the physical therapist based on results from the initial assessment, patient medical and treatment history, and presence of any functional limitations or comorbidities. |
| Murdoch et al., 2021  UK | Describe the organisational context of the hospital sites delivering the PREPARE-ABC exercise interventions, the nature of standard care, how staff organised intervention delivery within their particular care setting and how patients experienced the intervention. | Experimental (randomised control trial) | Y | Mixed methods | **S:** 14 UK hospitals.  **L:** Co-located or home exercise**.**  **C:** During-treatment.  **T:** Colorectal.  **D:** Three arms:   1) Hospital-supervised 2) Home-supported exercise 3) Treatment as usual   Participants randomised to one of the exercise interventions attended an initial 45-min counselling session at their treating hospital to initiate this process of self-determination, followed by face-to-face exercise sessions in the hospital-supervised arm, and motivational telephone calls in the home-supported arm.  Patients in the hospital-supervised arm were asked to attend up to three aerobic interval exercise sessions per week on a cycle ergometer over the 3–4weeks prior to surgery, plus two home-based resistance exercise sessions per week using resistance bands.  Patients randomised to the home supported arm were asked to comply with current public health physical activity guidelines [8] by engaging in at least 150 min of moderate-vigorous intensity aerobic exercise per week (e.g., brisk walking/jogging/cycling/ swimming), as well as completing two weekly sessions of resistance exercise using resistance bands. Six weeks after surgery, patients in the hospital-supervised arm were offered monthly “booster” supervised exercise sessions at the hospital, whilst those in the home-supported arm received monthly 15min telephone counselling calls to support continued engagement in their programme. |
| Pitcher et al., 2023  Australia | Evaluate a gym-based exercise program for patients with breast cancer from the perspectives and experiences of patients and breast care clinicians. | Pre-experimental | N | Mixed methods | **S:** Western Health, a large metropolitan public health service in the western suburbs of Melbourne, Australia.  **L:** Co-located.  **C:** During-treatment.  **T:** Breast.  **D:** Fully subsidised program provides patients at the end of their breast cancer treatment access to a supervised gym-based exercise program once a week for 12 weeks. Each participant is provided with an individualised program of aerobic and resistance exercises, the program is offered in a group setting. program is facilitated by a qualified personal trainer and funded by the health service’s charitable foundation. |
| Powell et al., 2023  UK | To understand how patients with colorectal, lung or oesophago-gastric cancer perceived a prehabilitation and recovery programme and to identify facilitators and barriers to engagement, whilst ensuring inclusion of individuals from lower socio-economic status areas. It also aimed to understand barriers and facilitators associated with referring patients to the programme, and the perspectives of healthcare staff on prehabilitation. | Pre-experimental | Y | Qualitative | **S:** National Health Service (NHS).  **L:** Referral to external service.  **C:** Across the continuum.  **T:** Mixed.  **D:** Greater Manchester Cancer Alliance Prehab4Cancer and Recovery Programme: Cancer care team (NHS) refer patients to P4C, which is run by GM Active: a collaboration of the public leisure providers in Greater Manchester. Patients’ fitness assessed, allocated to ‘universal’ or ‘targeted’ pathway.  ‘Targeted’ pathway: for individuals with lower fitness/greater support needs. Thrice-weekly sessions, supervised by Exercise Specialists  ‘Universal’ pathway: relatively independent, self-managed sessions in leisure facilities close to patient’s home, with monitoring by exercise specialists and support if needed. Both groups have free gym membership. |
| Reale et al., 2021  UK | Assess the acceptability and feasibility of embedding a supervised exercise pathway into standard National Health Service (NHS) prostate cancer care to meet current National Institute of Health and Care Excellence best practice recommendations. | Pre-experimental | N | Mixed methods | **S:** National Health Service (NHS).  **L:** Referral to external service.  **C:** During-treatment.  **T:** Prostate.  **D:** Supervised aerobic and resistance training were undertaken twice weekly for 12 weeks at Nuffield Health. The exercise prescription was tailored to account for the individual’s capability, opportunity and motivation and (where appropriate) any relevant medications and co-morbidities. Supervised exercise sessions were delivered face-to-face by an exercise trainer, one-to-one or in small groups (maximum, n = 5), with behavioural support (i.e. instruction, demonstration, prompting practise, feedback on behaviour, graded task). 30–45 min of moderate intensity aerobic exercise and up to four sets and 8–12 repetitions of resistance exercises targeting major muscle groups. Exercise intensity was set at between 65 and 85% of age predicted heart rate or 3 to 5 on the 10-point Borg rating of perceived exertion. |
| SantaMina et al., 2012  Canada | Describe our exercise intervention and integration strategy, and we offer insights about the facilitators and barriers to exercise program implementation within oncology. | Pre-experimental | Y | Qualitative | **S:** Comprehensive cancer treatment and research centre in downtown Toronto, Ontario, Canada.  **L:** Co-located.  **C:** During treatment.  **T:** Mixed.  **D:** Survivorship Exercise Program: multidisciplinary program that includes exercise physiologists, physicians, clinical psychologists, researchers, nurses, and student interns. Exercise programming is provided by certified exercise physiologists with oncology specific education and training. Exercise instruction and demonstration, and group exercise classes of 10–12 participants are held in an exercise room in an affiliated on-site cancer survivorship program. Also provided home exercise program. Participants are offered a variety of exercise-related services depending on their needs assessment and interests. |
| SantaMina et al., 2018  Canada | Describe our implementation pathway to integrating exercise information in the electronic medical record (EMR) at three cancer centres in North America. | Pre-experimental | Y | Qualitative | **Case A**  **S:** University of Alabama at Birmingham Cancer Centre**.**  **L:** Co-located.  **C:** NR.  **T:** NR.  **D:** The first effort to connect oncology care with qualified exercise professionals occurred via the already established and institutionally supported Weight Loss Medicine clinic. Using the ongoing health system infrastructure and identified information technology staff, a mechanism was created allowing oncology health care providers to refer overweight and obese cancer survivors interested in weight loss to the clinic via the EMR’s standard referral processes. Once seen by the weight loss medicine physician, patients are assisted with identifying appropriate qualified exercise professional, primarily outside the EMR system. In this respect, the EMR facilitated the integration of exercise services for cancer survivors via the weight loss medicine clinic because resources for new, large-scale, cancer-specific initiatives were not available. Several master’s trained exercise physiologists were certified as American College of Sports Medicine Cancer Exercise Trainers, and exercise sessions (individual and group) were made available on a fee for-service basis.  **Case B**  **S:** Sylvester Comprehensive Cancer Centre.  Co-located.  **C:** NR.  **T:** NR.  **D:** The Sylvester Comprehensive Cancer Centre launched an initiative to transfer all medical records across the institution to a new EMR system. The “GoLive” initiative provided a unique opportunity to leverage the university and hospital-wide mandate to substantiate and prioritise the inclusion of Oncology Support services with the new EMR. Qualified exercise professionals provide individualised activity counselling to patients with the option of enrolling patients in an 8-wk supervised exercise program offered at the campus’ medical wellness centre. Visits with the qualified exercise professionals are provided as a complementary service to standard care and at no cost to the patient (i.e., costs covered by local cancer centre). |
| SantaMina et al., 2018  Canada  SantaMina et al., 2019  Canada | Describe our implementation pathway to integrating exercise information in the electronic medical record (EMR) at three cancer centres in North America.  Describe the development and implementation of the program and reports on feasibility and effectiveness outcomes after a pilot and roll-out phase over nearly 4 years. | Pre-experimental  Pre-experimental | Y  N | Qualitative  Mixed methods | **Case C within Santa Mina et al., 2018**. This case description within Santa Mina et al., 2018 focussed only on the facility-based program  **S:** Princess Margaret Caner Centre, Canada.  **L:** Referral to external service or co-located.  **C:** Post-treatment.  **T:** Mixed.  **D:** Survivors completed an initial consultation with a kinesiologist to review past health history, determine contraindications and considerations for exercise, and discuss exercise experience and related goals. Resulted in either:   1) Discharge for patients for whom guidance about a physically active lifestyle and general exercise recommendations was sufficient for independent practice. 2) Referral to a community-based, cancer-exercise program if attendance at the hospital was challenging or because of patient preference. 3) Enrolment into the Wellness and Exercise for Cancer Survivors Program at the Princess Margaret. |
| Schmitz et al., 2024  USA | Evaluate whether integrating a rehabilitation navigator and applying the EXCEEDS tool would be acceptable and feasible from the perspective of patients and clinical staff, as well as to assess the extent to which this approach would improve the proportion of patients referred to appropriate exercise or rehabilitation programming. | Pre-experimental | N | Quantitative | **S:** University of Pittsburgh Medical Centre Hillman Cancer Centre  **L:** Referral to external service or co-located.  **C:** During-treatment.  **T:** Mixed.  **D:** Moving Through Cancer Triage Program used the EXCEEDS tool. Patients would complete a survey where referral would be based on health profile.   1) Referral to community-based exercise programming (multiple program options) 2) Outpatient rehabilitation 3) Clinically supervised sessions. |
| Voland et al., 2023  Germany | Identify barriers and facilitators for the implementation of OnkoActiv network structures from the perspective of regional centres and certified community-based exercise programs. | Pre-experimental | Y | Mixed methods | **S:** OnkoAktiv network throughout Germany  **L:** Co-located or referral to external service.  **C:** NR.  **T:** NR.  **D:** The OnkoAktiv pathway:   1) Identification and screening of patients in regard of their physical activity level by health care professionals e.g., oncologists, nurses 2) Health care professional advises exercise to patients and refer to best available community-based exercise program 3) If needing further evaluation and clearance for exercise, referred to exercise specialist with regional OnkoAktiv centres (e.g., cancer centres)   OnkoAktiv counts 14 regional centres and 106 certified community-based exercise programs across Germany.  Exercise specialists are organized in regional OnkoAktiv centers (RE) located incomprehensive cancer centers in Germany. Every RE engages as a single coordinating institution that recruits patients, offers consultations (including risk assessment and triage), exercise recommendation and collaborates within multidisciplinary teams. RE provide access to certified community-based exercise programs through patient referral processes and build up their own regional network.  Community based exercise programs can be located in, for example, local gyms, rehabilitation centres, physical therapy practices, or municipal facilities. Within such institutions, exercise trainers provide comprehensive exercise supervision for cancer patients based on physical activity recommendations. They implement the OnkoAktiv quality indicators that assure high-quality exercise program execution. |
| Wilson et al., 2021  USA | NR | Pre-experimental | N | Qualitative | **S:** Beaumont Cancer Institute  **L:** Co-located.  **C:** Across the continuum.  **T:** Mixed.  **D:** Beaumont’s Survivorship Exercise and Wellness Program: every patient with cancer receives an individualized exercise session and these exercises are performed under the supervision of a licensed physical therapist assistant (with specialty cancer training) in a group setting. |

D: description, EP: exercise physiologist, Hr: hour, L: exercise service delivery location**,** Min: minutes, N: no, NA: not applicable, NR: not reported, S: healthcare setting, Survivor: refers to an individual living with, through, and beyond a cancer diagnosis, UK: United Kingdom, USA: United States of America, Y: yes.
