# Supplementary material for: Facilitators to the implementation of exercise into cancer care: a systematic review
Source: J Cancer Surviv. 2026 Jan 4;20(Suppl 2):2237–50. doi: 10.1007/s11764-025-01948-5 (PMC13744452; doi:10.1007/s11764-025-01948-5)
Supplement: Supplementary file 1 [file 11764_2025_1948_MOESM1_ESM.zip › Supplementary/Supplementary Table 5.docx]

**Supplementary Table 5.** Stakeholder characteristics of included reports by implementation effort

Facilitators to the implementation of exercise into cancer care: a systematic review. Journal of Cancer Survivorship. Georgia L. White, Mary A. Kennedy, Brent Cunningham, Riley Dunn, Jason Martin, Meegan Walker, Craig T. Wallington-Gates, Hattie Wright, Bryan Chan, Grace L. Rose

**Correspondence:**

Georgia L White

University of the Sunshine Coast, Queensland, Australia

E-mail: georgia.white@research.usc.edu.au

| Study | Stakeholders | Stakeholder demographics |
| --- | --- | --- |
| Author  Year  Country |  | **S:** sex (females)  **A:** age (mean±SD)  **C:** cancer type/stage (people living with or beyond cancer only) |
| Adsul et al., 2022  USA | **Case A**  NA | **S:** NA (no stakeholders included within report)  **A:** NA (no stakeholders included within report)  **C:** NA (no stakeholders included within report) |
| Adsul et al., 2022  USA  Beidas et al., 2014  USA | **Case C**  NA  Deliverers (physiotherapists) (n=10) | **S:** NA (no stakeholders included within report)  **A:** NA (no stakeholders included within report)  **C:** NA (no stakeholders included within report)  **S:** n=16, 86% (only reported across n=19 sample size)  **A:** NR  **C:** NA |
|  | Referrers (n=9)  *Nurse practitioners n=2*  *Physicians n=7* | **S:** n=16, 86% (only reported across n=19 sample size)  **A:** NR  **C:** NA |
| Barnes et al., 2020  USA | NA | **S:** NA (no stakeholders included within report)  **A:** NA (no stakeholders included within report)  **C:** NA (no stakeholders included within report) |
| Brennan et al., 2024  Ireland | NA | **S:** NA (no stakeholders included within report)  **A:** NA (no stakeholders included within report)  **C:** NA (no stakeholders included within report) |
| Caperchione et al., 2023  Australia | Referrers (oncologists/haematologists) (n=3) | **S:** NR  **A:** NR  **C:** NA |
|  | Deliverers (EP) (n=1)  **People living with or beyond cancers not extracted as per scoping review inclusion criteria (people living with or beyond cancers did not have influence over implementation outcomes** | **S:** NR  **A:** NR  **C:** NA |
| Czosnek et al., 2023  Australia | **Case A**  Deliverers (EP n=1)  Referrers, organisational (n=4)  *Program manager n=1, referral source n=1, other allied health staff n=1, executive n=1*  Observations (n=40 hrs)  **Case B not extracted as per scoping review inclusion criteria** | **S:** NR  **A:** NR  **C:** NA  **S:** NA  **A:** NA  **C:** NA |
|  | **Case C**  Deliverers (EP n=3)  Referrers, organisational (n=5)  *Program manager n=2, referral source n=1, other allied health staff, n=2*  Observations (n=15 hrs) | **S:** NR  **A:** NR  **C:** NA  **S:** NA  **A:** NA  **C:** NA |
| Dalzell et al., 2017  Canada | NA | **S:** NA (no stakeholders included within report)  **A:** NA (no stakeholders included within report)  **C:** NA (no stakeholders included within report) |
| Dennett et al., 2017  Australia | Deliverers (n=31)  *Public n=14, private n=17* | **S:** NA  **A:** NA  **C:** NA |
| Dennett et al., 2021  Australia | Deliverers and organisational (n=8)  *Physiotherapists n=3, nurse n=1, allied health assistant n=1, administration staff n=2, manager n=1*  **People living with or beyond cancers not extracted as per scoping review inclusion criteria (people living with or beyond cancers did not have influence over implementation outcomes** | **S:** NR  **A:** NR  **C:** NA |
| Dennett et al., 2021  Australia  Dennett et al., 2021  Australia | Deliverers (physiotherapists n=2)  Referrers (n=15)  *Oncologists n=2, nurses n=7, occupational therapist n=1, social worker n=1, physiotherapists [not involved in delivery] n=2*  **People living with or beyond cancers not extracted as per scoping review inclusion criteria (people living with or beyond cancers did not have influence over implementation outcomes** | **S:** n=13, 87%  **A:** NR  **C:** NA  **S:** NA  **A:** NA  **C:** NA |
| Devenney et al., 2023  Ireland | Referrers (professions NR) (n=8)  **People living with or beyond cancers not extracted as per scoping review inclusion criteria (people living with or beyond cancers did not have influence over implementation outcomes** | **S:** NR  **A:** NR  **C:** NA |
| Faro et al., 2023  USA | Phase 1: Referrers (n=4)  *Physician’s assistant n=1, nurse practitioners n=3*  **People living with or beyond cancers not extracted as per scoping review inclusion criteria (people living with or beyond cancers did not have influence over implementation outcomes** | **S:** NR  **A:** NR  **C:** NR |
| Ijsbrandy et al., 2020  Netherlands | Primary healthcare professionals; deliverers and referrers (n=31)  *General practitioners within hospitals n=14, physiotherapists n=17* | **S:** n=10, 52%  **A:** 48±11  **C:** NA |
|  | Secondary healthcare professionals; referrers (n=39)  *Physician/surgeon n=13, paramedic n=12, nurse n=13, other n=1* | **S:** n=32, 82%  **A:** 43±11  **C:** NA |
| Kennedy et al., 2020  Australia | Referrers (n=15)  *Radiation oncologists n=7, nurses n=8* | **S:** NR  **A:** NR  **C:** NA |
|  | Deliverers (EPs) (n=3)  **People living with or beyond cancers not extracted as per scoping review inclusion criteria (people living with or beyond cancers did not have influence over implementation outcomes** | **S:** NR  **A:** NR  **C:** NA |
| Kennedy et al., 2022  Australia | Exercise working group (n=10)  *Organisational leaders n=2, clinicians n=1, people living with or beyond cancers n=2, researchers n=4, implementation adviser n=1*  **People living with or beyond cancers [that were not included within the working group] not extracted as per scoping review inclusion criteria (people living with or beyond cancers did not have influence over implementation outcomes** | **S:** NR  **A:** NR  **C:** NR |
| Kim et al., 2024  USA | Referrers (n=12)  *Oncology providers n=3, registered nurses n=3, medical assistant/undergraduate medical assistant interns/pharmacy tech/ n=4, receptionists n=2* | **S:** n=6, 50%  **A:** NR  **C:** NA |
| Leach et al., 2018  USA | NA | **S:** NA (no stakeholders included within report)  **A:** NA (no stakeholders included within report)  **C:** NA (no stakeholders included within report) |
| Murdoch et al., 2021  UK | Deliverers (n=13)  *Research practitioners n=9, physiotherapists n=3 and exercise practitioner n=1* | **S:** NR  **A:** NR  **C:** NA |
|  | Observations by research team  *Observations of intervention arms n=14, standard care observations n=34*  **People living with or beyond cancers not extracted as per scoping review inclusion criteria (people living with or beyond cancers did not have influence over implementation outcomes** | **S:** NA  **A:** NA  **C:** NA |
| Pitcher et al., 2023  Australia | Referrers (n=4)  *(Medical oncologists n=2, breast care nurse consultants n=2)*  **People living with or beyond cancers not extracted as per scoping review inclusion criteria (people living with or beyond cancers did not have influence over implementation outcomes** | **S:** NR  **A:** Mean NR, (range 38-55)  **C:** NA |
| Powell et al., 2023  UK | Referrers/involved in referral process (n=24)  *Nurses n=11, doctors n=7, other n=4, NR n=2* | **S:** n=16, 67%  **A:** Median 44 years (range: "30s-50s")  **C:** NA |
| Reale et al., 2021  UK | Referrers (professions NR) (n=8)  **Community interventionists not extracted as per scoping review inclusion criteria**  **People living with or beyond cancers not extracted as per scoping review inclusion criteria (people living with or beyond cancers did not have influence over implementation outcomes** | **S:** NR  **A:** NR  **C:** NA |
| SantaMina et al., 2012  Canada | NA | **S:** NA (no stakeholders included within report)  **A:** NA (no stakeholders included within report)  **C:** NA (no stakeholders included within report) |
| SantaMina et al., 2018  Canada | NA | **Case A**  **S:** NA  **A:** NA  **C:** NA  **Case B**  **S:** NA  **A:** NA  **C:** NA |
| SantaMina et al., 2018  Canada  SantaMina et al., 2019  Canada | NA | **S:** NA (no stakeholders included within report)  **A:** NA (no stakeholders included within report)  **C:** NA (no stakeholders included within report)  **S:** NA (no stakeholders included within report)  **A:** NA (no stakeholders included within report)  **C:** NA (no stakeholders included within report) |
| Schmitz et al., 2024  USA | NA | **S:** NA (no stakeholders included within report)  **A:** NA (no stakeholders included within report)  **C:** NA (no stakeholders included within report) |
| Voland et al., 2023  Germany | Regional OnkoAktiv Centre deliverers (n=6)  *Only cancer care clinics extracted*  **Community interventionists not extracted as per scoping review inclusion criteria**  **Facilitators relating only to cancer care clinics were extracted as per scoping review inclusion criteria** | **S:** n=6, 75% (percentage relative to the n=8 reported Regional OnkoAktiv Centre professionals)  **A:** NR  **C:** NA |
| Wilson et al., 2021  USA | NA | **S:** NA (no stakeholders included within report)  **A:** NA (no stakeholders included within report)  **C:** NA (no stakeholders included within report) |

A: age, C: cancer type and stage, EP: exercise physiologist, NA: not applicable, NR: not reported, S: sex (females), UK: United Kingdom, USA: United States of America.
