# Supplementary material for: Facilitators to the implementation of exercise into cancer care: a systematic review
Source: J Cancer Surviv. 2026 Jan 4;20(Suppl 2):2237–50. doi: 10.1007/s11764-025-01948-5 (PMC13744452; doi:10.1007/s11764-025-01948-5)
Supplement: Supplementary file 1 [file 11764_2025_1948_MOESM1_ESM.zip › Supplementary/Supplementary Table 6.docx]

**Supplementary Table 6.** Facilitators by included implementation effort

Facilitators to the implementation of exercise into cancer care: a systematic scoping review. Journal of Cancer Survivorship. Georgia L. White, Mary A. Kennedy, Brent Cunningham, Riley Dunn, Jason Martin, Meegan Walker, Craig T. Wallington-Gates, Hattie Wright, Bryan Chan, Grace L. Rose

**Correspondence:**

Georgia L White

University of the Sunshine Coast, Queensland, Australia

E-mail: georgia.white@research.usc.edu.au

| Implementation effort | I | OS | IS | IN | IP |
| --- | --- | --- | --- | --- | --- |
| Adsul et al., 2022  USA |  |  |  | 2 | 4 |
| Adsul et al., 2022  USA  Beidas et al., 2014  USA | 1 |  | 1 | 1 | 3 |
| Barnes et al., 2020  USA |  |  |  | 1 | 2 |
| Brennan et al., 2024  Ireland | 1 | 1 |  | 1 |  |
| Caperchione et al., 2023  Australia | 2 |  |  |  | 2 |
| Czosnek et al., 2023  Australia  Case A  Case C | 4  4 | 2  2 | 5  5 | 2  1 | 2  2 |
| Dalzell et al., 2017  Canada | 1 |  | 2 |  | 1 |
| Dennett et al., 2017  Australia | 4 |  |  | 1 | 2 |
| Dennett et al., 2021  Australia | 2 |  | 1 | 1 |  |
| Dennett et al., 2021  Australia  Dennett et al., 2021  Australia | 7 | 1 | 6 | 2 | 3 |
| Devenney et al., 2023  Ireland | 1 |  |  | 1 | 1 |
| Faro et al., 2023  USA | 1 |  | 2 |  | 3 |
| Ijsbrandy et al., 2020  Netherlands | 1 |  |  |  | 1 |
| Kennedy et al., 2020  Australia |  |  |  | 1 |  |
| Kennedy et al., 2022  Australia | 1 | 1 | 4 | 1 | 3 |
| Kim et al., 2024  USA |  |  | 2 | 1 | 2 |
| Leach et al., 2018  USA |  |  | 1 |  |  |
| Murdoch et al., 2021  UK | 1 |  |  |  | 1 |
| Pitcher et al., 2023  Australia | 1 |  |  |  |  |
| Powell et al., 2023  UK |  |  |  | 1 | 2 |
| Reale et al., 2021  UK | 1 |  | 1 | 3 |  |
| SantaMina et al., 2012  Canada | 1 | 2 | 1 |  | 1 |
| SantaMina et al., 2018  Canada  Case A  Case B |  |  | 1  3 | 1 |  |
| SantaMina et al., 2018  Canada  (Case C)  SantaMina et al., 2019  Canada |  |  | 4 |  | 2 |
| Schmitz et al., 2024  USA |  |  | 2 |  | 2 |
| Voland et al., 2023  Germany |  | 1 | 3 | 1 | 1 |
| Wilson et al., 2021  USA | 1 | 1 | 5 | 2 | 5 |
| TOTAL | **35** | **11** | **49** | **24** | **45** |
| Average number of facilitators per implementation effort n = 6 | | | | | |
| Average number of CFIR domains per implementation effort n = 3 | | | | | |

CFIR: Consolidated Framework for Implementation Research, I: innovation, OS: outer setting, IS: inner setting, IN: individuals, IP: implementation process
